# A Rare Case of Thoracic SMARCA4-Deficient Undifferentiated Tumor With Diffuse Brain Metastasis

**DOI:** 10.7759/cureus.61367

**Published:** 2024-05-30

**Authors:** Shui Ho Chan, Lei Alena M Dagat, Naeem Latif

**Affiliations:** 1 Family Medicine, University of Pittsburgh Medical Center Lititz, Lititz, USA; 2 Internal Medicine, University of Pittsburgh Medical Center Lititz, Lititz, USA; 3 Hematology and Oncology, University of Pittsburgh Medical Center Lititz, Lititz, USA

**Keywords:** smarca4 mutation, multiple intracranial lesions, diffuse brain metastasis, smarca4-deficient undifferentiated tumor, thoracic tumor

## Abstract

Thoracic SMARCA4-deficient undifferentiated tumor (SMARCA4-UT) is a recently described rare and aggressive malignancy characterized by undifferentiated cell morphology and the loss of the Brahma-related gene 1 (BRG1) protein. Its pathogenesis involves mutational loss of SMARCA4 gene expression, which encodes the BRG1 protein that serves as one of the catalytic subunits of the SWItch/Sucrose Non-Fermentable (SWI/SNF) chromatin remodeling complex. This malignancy of the thorax predominantly affects middle-aged male smokers and commonly metastasizes to lymph nodes, bones, adrenal glands, liver, gastrointestinal tract, central nervous system, and kidney. Cases of brain metastasis have been reported but are less common. We report a case of this tumor initially presenting with diffuse brain metastasis in a 55-year-old male with a significant smoking history. We reviewed the current literature on the diagnostic and therapeutic challenges posed by this highly aggressive thoracic tumor.

## Introduction

Thoracic SMARCA4-deficient undifferentiated tumor (SMARCA4-UT) was first described in 2015 by Le Loarer et al., who identified a group of rare, distinct thoracic malignancies which they named SMARCA4-deficient thoracic sarcoma (SMARCA4-DTS) [1]. The name was later changed to thoracic SMARCA4-UT in the 2021 WHO Classification of Thoracic Tumors [2]. The pathogenesis of thoracic SMARCA4-UT involves the mutational loss of expression of the *SMARCA4* gene located on chromosome 19p, which encodes the Brahma-related gene 1 (BRG1) protein, one of the two mutually exclusive catalytic subunits of the mammalian SWItch/Sucrose Non-Fermentable (SWI/SNF) ATP-dependent chromatin remodeling complex [3,4]. The diagnosis of thoracic SMARCA4-UT is made based on clinical findings of a thoracic mass with poorly differentiated small cell, epithelioid, or rhabdoid appearance in morphology and hallmark loss of BRG1 protein expression [2,3]. SMARCA4 deficiency is occasionally seen in conventional non-small cell lung carcinoma (NSCLC). However, SMARCA4-deficient NSCLC (SMARCA4‐dNSCLC) typically has differentiated squamous or glandular histology and is recognized as a separate tumor entity from SMARCA4-UT under the WHO Thoracic Tumor Classification [5]. Clinical signs and symptoms of this tumor are similar to other malignancies that involve the thorax, including dyspnea, chest pain, regurgitation, fatigue, unintentional weight loss, appetite change, and local or systemic symptoms determined by the site of distal metastasis [1-3]. Thoracic SMARCA4-UT commonly metastasizes to lymph nodes, bones, adrenal glands, liver, gastrointestinal tract, central nervous system, and kidney [6-9]. Brain metastasis is less commonly reported compared to other sites of metastasis [4,6-9]. This case report describes a 55-year-old male who presented with diffuse multiple brain metastases and two left lung masses that were immunohistochemically confirmed to be thoracic SMARCA4-UTs.

## Case presentation

**Figure 1:**
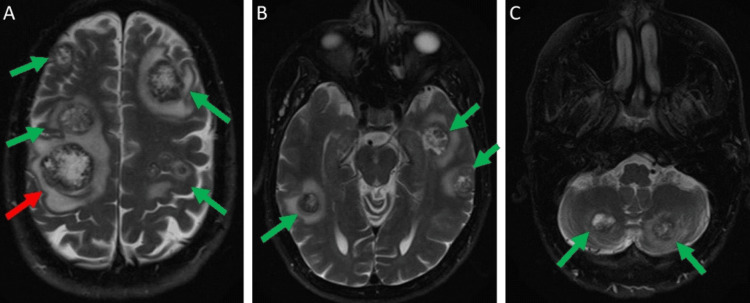
Initial MRI brain with IV contrast T2-weighted images. (A) Multiple brain lesions (green arrows) with significant surrounding edema involving bilateral frontal and parietal lobes, with the largest lesion measuring 2.9 cm in the right frontal lobe (red arrow). (B) Multiple brain lesions with surrounding edema (green arrows) involving bilateral temporal lobes. (C) Brain lesions (green arrows) with surrounding edema involving bilateral cerebellum. MRI: magnetic resonance imaging; IV: intravenous.

A 55-year-old Caucasian male with a past medical history significant only for nicotine abuse presented with a three-week history of neurological symptoms, including left-sided facial droop, left upper extremity weakness, and sporadic difficulty reading text messages and operating vehicles. The patient otherwise did not have any respiratory or cardiovascular symptoms. He had normal constitutional, pulmonary, cardiovascular, musculoskeletal, and abdominal physical examination findings on presentation. He ambulated independently prior to the initial evaluation. Pertinent social history included employment as a truck driver and being a heavy, active smoker of three packs per day for the past 30 years. His family history of cancer consisted of breast cancer, with his mother diagnosed in her 40s, and head and neck cancer, with his sister in her 30s. His initial computed tomography (CT) and magnetic resonance imaging (MRI) of the brain with intravenous (IV) contrast revealed multiple large enhancing parenchymal lesions associated with parenchymal edema throughout the brain (Figure 1A-C). The largest lesion measured approximately 2.9 cm in the posterior right frontal lobe (Figure 1A).

**Figure 2:**
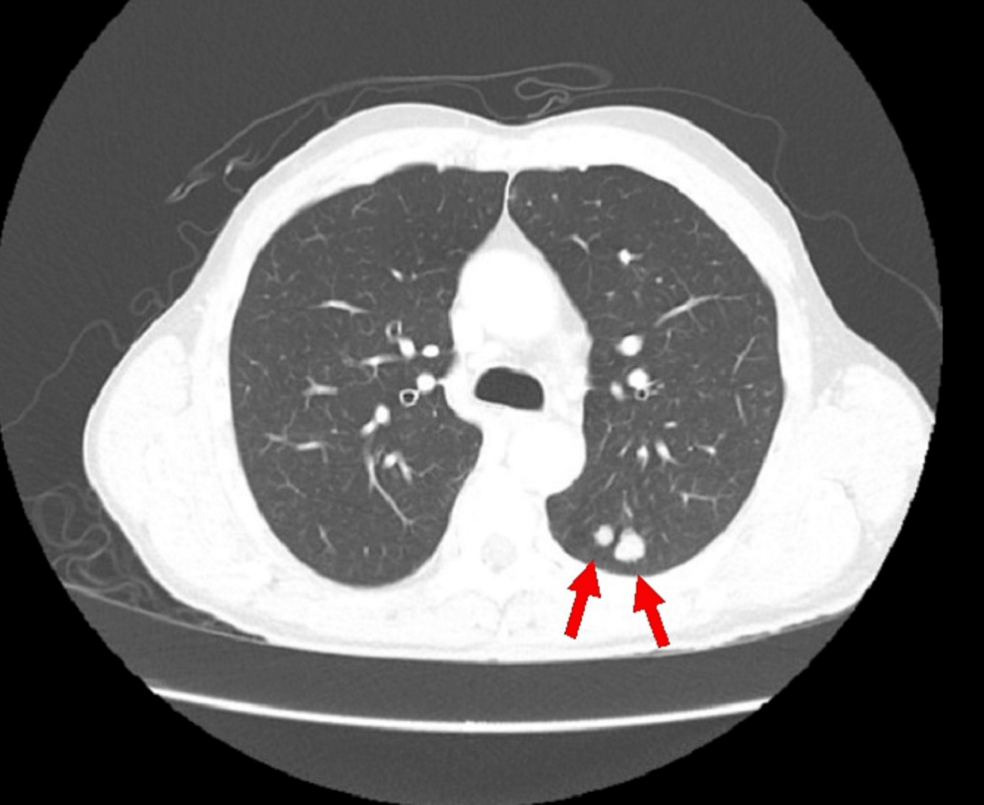
Initial CT chest with IV contrast. CT chest showing two left posterior pulmonary nodules measuring 7 and 12 mm in size (red arrows). CT: computed tomography; IV: intravenous.

His CT chest, abdomen, and pelvis with IV contrast demonstrated two non-calcified pulmonary nodules in the posterior left upper lobe, measuring approximately 7 and 12 mm, and associated with left hilar adenopathy (Figure 2). His initial CT imaging did not show any suspicious spread or lesion in the abdominal or pelvic region. The patient’s blood work, including complete blood count, basic metabolic panel, clotting profile, and common tumor markers (CEA, AFP, CA19-9, PSA, CA15-3), was within normal limits (Appendix Table 1).

**Figure 3:**
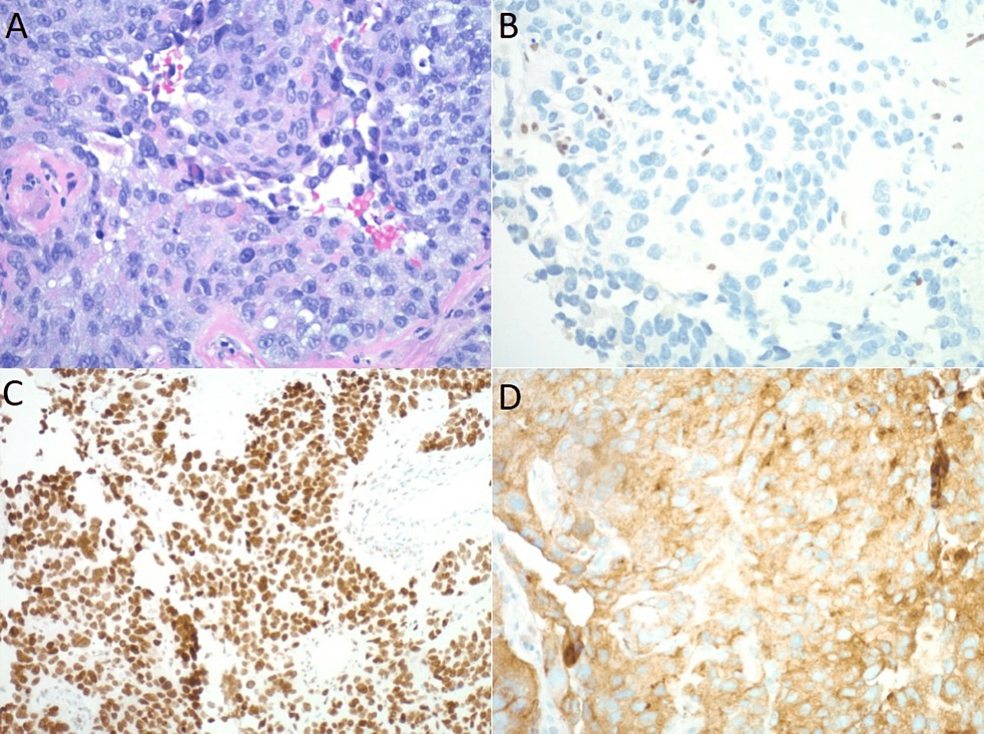
Histopathology of the pulmonary nodule. (A) H&E stain (20x) showing poorly differentiated malignant tumor. (B) Immunohistochemical staining negative for BRG1 (20x). (C) Immunohistochemical staining positive for SOX2 (20x). (D) Immunohistochemical staining positive for synaptophysin (20x). H&E: hematoxylin and eosin; BRG1: Brahma-related gene 1.

CT-guided biopsy of the left upper lobe pulmonary nodule was subsequently performed, resulting in a histologically poorly differentiated malignant neoplasm (Figure 3A). Immunohistochemistry (IHC) stains of the tissue determined the tumor was BRG1 negative, SOX2 positive, and synaptophysin positive (Figure 3B-D). Other markers tested include INSM1 negative, P40 negative, CK20/TTF1 dual stain negative, CK7/CDX2 dual stain negative, NKX3.1 negative, PAX8 negative, Napsin A negative, MelanA negative, PLAP negative, Ki-67 greater than 80%, and AE1/AE3 rare and focal positivity. The above findings confirm the diagnosis of SMARCA4-UT, given the poorly differentiated morphology and the lack of expression of BRG1. Positive expression of SOX2 and synaptophysin are common findings of this tumor and support the diagnosis [2,8,10]. 

The patient was diagnosed with clinical Stage IVB neoplasm of the left lung with brain metastasis. He was evaluated by neurosurgery and considered not a surgical candidate given his diffuse brain metastasis. He was discharged from the hospital and maintained on dexamethasone 4 mg twice daily and lamotrigine 750 mg twice daily for seizure precaution. He received whole brain radiation for 10 treatment fractions of 3.0 Gy per fraction, for a total dose of 30.0 Gy over 13 days. He was then started on chemotherapy consisting of carboplatin and paclitaxel two weeks after the completion of brain radiation. 

**Figure 4:**
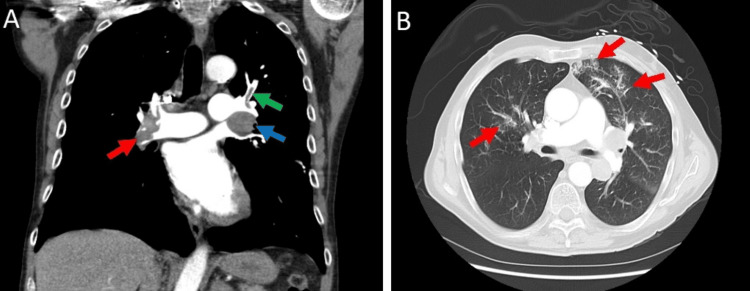
CTPE protocol of the chest. (A) Contrast-enhanced angiogram showing a large thrombus at the right main pulmonary artery (red arrow) and a thrombus on the left upper branch of the pulmonary artery (green arrow). There is also enlarged left hilar lymphadenopathy measuring up to 2.5 x 3.3 cm (blue arrow). (B) Contrast-enhanced angiogram showing ground glass opacities bilateral lungs (red arrow) suspicious of pulmonary infarcts or pulmonary hemorrhages. CTPE: computed tomography pulmonary embolism.

**Figure 5:**
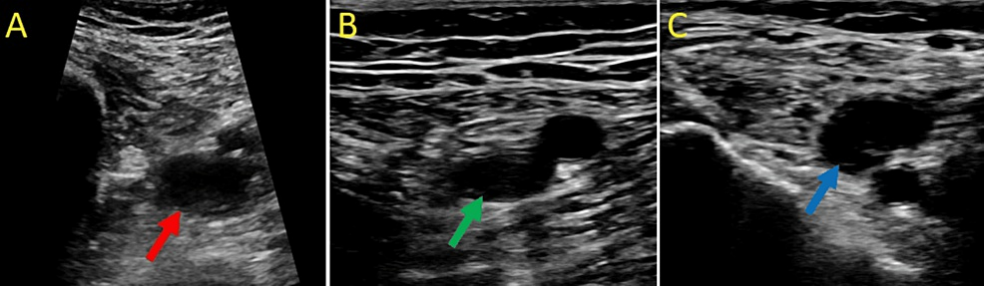
Ultrasound of the right lower extremity with incompressible veins with a transducer probe. (A) Non-compressible right proximal femoral vein (red arrow), (B) non-compressible right distal femoral vein (green arrow), and (C) non-compressible right popliteal vein (blue arrow). Non-compressible veins indicate the presence of venous thrombi, and these findings are consistent with DVT of the right lower extremity. DVT: deep vein thrombosis.

Two weeks after his first cycle of chemotherapy, he was admitted to the hospital for respiratory distress with hypoxia. CT pulmonary embolism (CTPE) protocol of the chest revealed bilateral pulmonary emboli (Figure 4A) and bilateral patchy ground glass opacities suspicious of pulmonary infarcts (Figure 4B). Doppler ultrasound revealed right lower extremity deep vein thrombosis (DVT) at multiple veins (Figure 5A-C). Discussion with the Pulmonary Embolism Response Team (PERT) and the hematology/oncology team concluded that the patient was a poor candidate for thrombectomy and anticoagulation in the setting of his diffuse brain metastasis with a high risk for intracranial bleeding. The patient underwent an IVC filter placement and was discharged home in stable condition to continue his chemotherapy treatments. He has a new 3 L oxygen requirement for activity at home upon discharge. 

**Figure 6:**
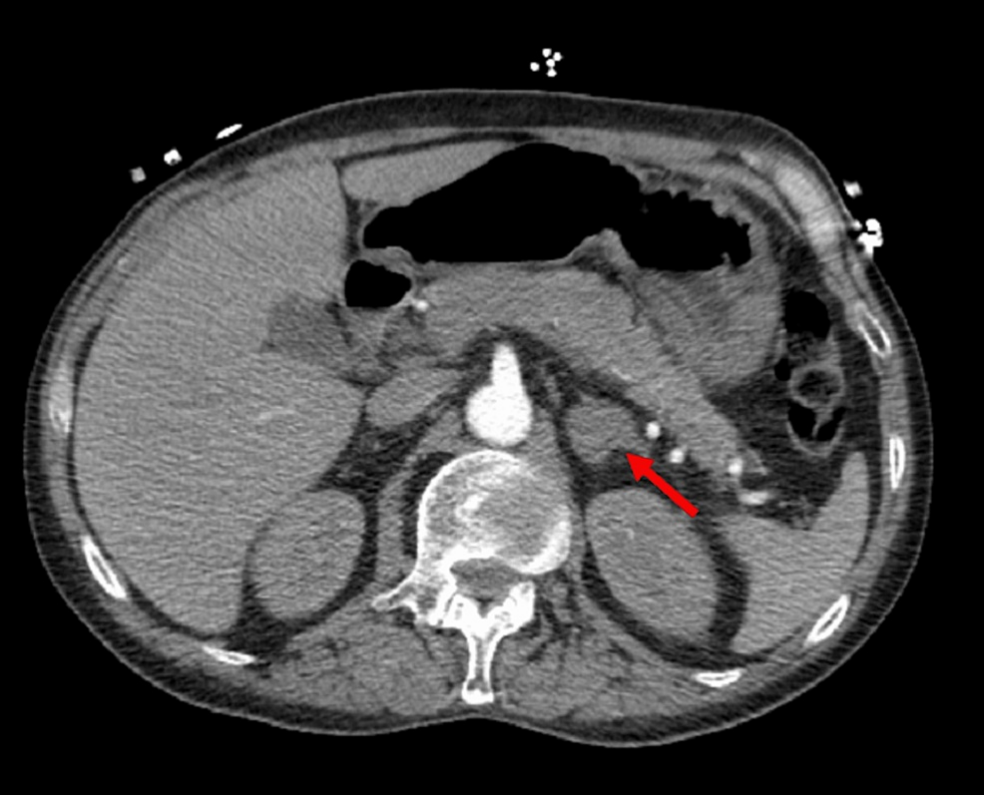
CT angiogram of the chest with contrast. CT chest, including parts of the upper abdomen, showing a new 2.7 cm enhancing left adrenal nodule not seen on his initial CT abdomen eight weeks prior, suspicious of metastasis. CT: computed tomography.

**Figure 7:**
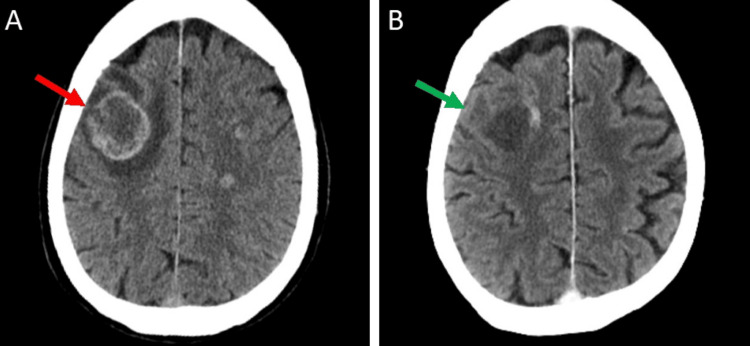
CT brain with IV contrast before and after whole brain radiation. (A) Initial CT brain with IV contrast showing a large parenchymal lesion at the right frontal lobe (red arrow). (B) Follow-up CT brain with contrast four weeks after whole brain radiation, showing reduction in size and edema of the same lesion (green arrow). CT: computed tomography.

Comparing the CTPE scan to his initial CT chest scan eight weeks prior, the two pulmonary nodules were stable, but the associated left hilar and mediastinal lymph nodes had increased in size. This CTPE also revealed a new 2.7 cm enhancing left adrenal nodule suspicious for metastasis (Figure 6). His neurological symptoms, including left facial droop, left upper extremity weakness, and difficulty reading, did improve after whole brain radiation. His follow-up CT of the brain four weeks after whole brain radiation showed a decrease in the size of lesions and significant resolution of surrounding edema (Figure 7A, B). Nevertheless, given the poor prognosis of this tumor, the patient decided to stop further investigations and aggressive interventions and eventually signed with hospice just three months after this cancer diagnosis.

## Discussion

Thoracic SMARCA4-UT shares common clinical symptoms with other tumors involving the thorax, including dyspnea, chest pain, regurgitation, fatigue, unintentional weight loss, and appetite change. The location of the metastasis will also contribute to other symptoms such as neurological deficits and seizures from brain metastasis, localized pain and pathological fractures from bone metastasis, or jaundice and elevated liver enzymes from liver metastasis. It can be difficult to distinguish from other thoracic tumor diagnoses, and differential diagnoses include non-small cell lung cancer (NSCLC), small cell lung cancer (SCLC), SMARCA4-deficient NSCLC, breast cancer, renal cell carcinoma, melanoma, colorectal cancer, gastroesophageal cancer, thyroid cancer, thymic cancer, lymphoma, neuroendocrine carcinomas, malignant mesothelioma, NUT carcinomas, and other rare thoracic sarcomas [1-3]. Many other differential diagnoses can present with similar diffuse multiple intracranial lesions as well, including but not limited to glioblastoma multiforme, gliomatosis cerebri, primary central nervous system lymphoma (PCNSL), medulloblastoma, multiple sclerosis, sarcoidosis, acute disseminated encephalomyelitis, neurocysticercosis, toxoplasmosis, tuberculosis, anti-NMDA receptor encephalitis, and progressive multifocal leukoencephalopathy (PML) [11]. 

Thoracic SMARCA4-UT tumors primarily occur in the mediastinum of male smokers. These tumors are characterized by poorly differentiated cells, often with small cell, epithelioid, or rhabdoid appearance, expressing variable epithelial markers [3]. The somatic mutation of SMARCA4 and the loss of Brahma-related gene 1 (BRG1) protein are hallmarks of these tumors [1-3]. Other findings that support this diagnosis include loss of BRM, negative claudin-4, and positive expression of SOX2, CD34, and SALL4 [2-3,9]. Synaptophysin is also commonly found to be positive in these tumors in up to 70% of cases [8,10]. The *SMARCA4* gene located on chromosome 19p encodes the BRG1 protein, one of the two mutually exclusive catalytic subunits of the mammalian SWI/SNF ATP-dependent chromatin remodeling complex [12]. The SWI-SNF complex is highly evolutionarily conserved and plays a vital role in nucleosome positioning, transcription, replication, DNA repair and recombination, regulation of gene expression, and cell cycle regulation [12,13]. Somatic mutations in genes encoding for various subunits of the SWI/SNF complex have been found in many adult-onset malignancies [3,9,12,13]. SMARCA4 deficiency is also found in NSCLC but is considered a separate disease due to differences in tissue morphology, RNA expression, and prognosis [8]. While SMARCA4-UT tumors share some similarities with SMARCA4-deficient NSCLC, they exhibit distinct genetic and morphological features more akin to other tumors like malignant rhabdoid tumor (MRT) and high-boundary small-cell carcinoma of the ovary of the hypercalcemic type (SCCOHT) [1,2]. Hence, diagnosis of thoracic SMARCA4-UT requires incorporating immunohistochemical testing of the tumor cells, and arriving at the correct diagnosis is a crucial step in guiding prognosis and treatment choice.

Treatment guidelines for thoracic SMARCA4-UT are lacking, and research on the effectiveness of chemotherapy is limited. A typical chemotherapy regimen involves various combinations of paclitaxel, carboplatin, gemcitabine, cisplatin, and doxorubicin [2]. The effectiveness of chemotherapy is unclear due to the low prevalence of identified cases and the highly aggressive nature of the tumor. There has been a report of a surgically resected case that achieved remission after nine months [14]. However, this tumor is known to have a high rate of recurrence, with multiple reported surgically resected cases showing recurrence within one month [1,15]. The prognosis for patients with thoracic SMARCA4-UT is generally poor, with a reported median survival of four to seven months [6]. The reported two-year overall survival rate is low at 12.5% [4]. Early palliative care involvement facilitates advanced care planning and allows patients to express their preferences regarding end-of-life care for this highly aggressive tumor entity. For our case, carboplatin and paclitaxel were initially chosen in the setting of not knowing programmed death ligand 1 (PD-L1) expression status, given that the literature had shown treatment-responsive cases that utilized both of those agents [16]. Unfortunately, due to early complications and the poor prognosis of this tumor, our patient decided to stop chemotherapy after just one cycle, without proceeding with further molecular testing that could potentially guide immunotherapy for his cancer.

Immunotherapy and targeted therapy modalities are currently under investigation and have shown promising efficacy [17]. In particular, immune checkpoint inhibitors (ICIs) targeted against programmed cell death protein 1 (PD-1) and PD-L1, such as pembrolizumab and nivolumab, have demonstrated treatment response in several case studies of thoracic SMARCA4-UT. Takada et al. reported a case with 60% of tumor cells expressing PD-L1, showing treatment response to pembrolizumab treatment [7]. Pokhrel et al. similarly reported a case with a PD-L1 proportional score of 45-50%, showing treatment response after three cycles of carboplatin and paclitaxel and one cycle of pembrolizumab [16]. In contrast, Henon et al. reported a case with negative PD-L1 that was also responsive to pembrolizumab [18]. Naito et al. and Iijima et al. each reported a case of SMARCA4-UT that responded to nivolumab with sustained tumor regression for 14 months and 22 months, respectively [19,20]. Both of those cases had a high tumor mutation burden (TMB) and low PD-L2 expression. While the number of cases is small, these cases suggest that the thoracic SMARCA4-UT treatment response to ICI does not necessarily correlate with PD-L1 expression. Targeted inhibitor therapy against Enhancer of Zeste Homolog 2 Histone Methyltransferase (EZH2) has also been proposed based on evidence of treatment response to other tumors with similar pathogenesis of SWI/SNF complex altering mutations, including malignant rhabdoid tumors and small cell carcinomas of the ovary hypercalcemic type [10].

## Conclusions

This case report depicts a rare diffuse brain metastasis presentation of thoracic SMARCA4-UT. As a single case report, this study has limitations regarding generalizability to the population and inability to establish any causality between risk factors and this cancer. This study also lacks the statistical power to determine the treatment response and the significance of the observed findings in this patient. Our study does echo other thoracic SMARCA4-UT cases in the literature regarding the highly aggressive nature of this tumor and poor treatment response to conventional chemotherapy alone. Ongoing research is warranted to expand our understanding and guide optimal treatment strategies for this novel and highly aggressive tumor entity. Treatment options being explored, such as ICIs and targeted inhibitor therapy, offer hope for future patients facing this challenging diagnosis. ICIs targeting PD-1 and PD-L1 have shown promising treatment potential in the literature, guiding future research for thoracic SMARCA4-UT.

## Appendices

**Table 1:** Laboratory testing result on initiation presentation. Normal lab results of CBC, BMP, clotting profile, and tumor markers. BMP: basic metabolic panel; CC: complete blood count; MCV: mean corpuscular volume; MCH: mean corpuscular hemoglobin; MCHC: mean corpuscular hemoglobin concentration; MPV: mean platelet volume; RDW: red cell distribution width; eGFRcr: creatinine-based estimated glomerular filtration rate; PT: prothrombin time; PTT: partial thromboplastin time; INR: international normalized ratio; CEA: carcinoembryonic antigen; AFP: alpha-fetoprotein; PSA: prostate-specific antigen.

CBC (reference range)	
WBC count	
(3.9-9.5 K/µL)	7
RBC count	
(4.17-5.63 M/µL)	4.82
Hemoglobin	
(12.8-16.6 G/DL)	16.2
Hematocrit	
(38.0-49.0%)	48.4
MCV	
(82.1-100.6 FL)	100.5
MCH	
(26.5-33.7 PG)	33.5
MCHC	
(31.8-36.0 G/DL)	33.4
Platelet count	
(140-366 K/µL)	159
MPV	
(6.6-12.2 FL)	9.9
RDW	
(11.8-14.9%)	13.7
Neutrophils	
(47.1-77.6%)	70.6
Lymphocytes	
(15.7-41.2%)	20.3
Monocytes	
(1.0-11.5%)	8.3
Eosinophils	
(0.0-7.3%)	0.3
Basophils	
(0.0-1.5%)	0.5

Basic metabolic profile (reference range)	
Sodium	
(136-145 mmol/L)	140
Potassium	
(3.5-5.1 mmol/L)	3.8
Chloride	
(98-107 mmol/L)	104
CO_2_	
(19-33 mmol/L)	28
Urea nitrogen, blood	
(7-25 mg/dL)	15
Creatinine	
(0.70-1.30 mg/dL)	0.87
Glucose	
(70-99 mg/dL)	97
Calcium	
(8.6-10.3 mg/dL)	9.5
Anion Gap	
(5.0-11.0 mmol/L)	8
eGFRcr	
(≥60 mL/min/1.73 SQM)	102

Clotting profile	
PT	
(9.4-12.5 seconds)	12.1
PTT	
(25.1-36.5 seconds)	27.7
INR	
(0.8-1.1)	1

Tumor markers (reference range)	
CEA	
(<5 ng/mL for smokers)	2.6
AFP tumor marker	
(<6.1 ng/mL)	3.1
Ca-19-9(Tm)	
(<34 U/mL)	8
Total PSA	
(≤4.0 ng/mL)	0.5
Ca 15-3	
(<32 U/mL)	9
